# Performance Enhancement and Applications Review of Nano Light Emitting Device (LED)

**DOI:** 10.3390/nano11010023

**Published:** 2020-12-24

**Authors:** Harel Perlman, Tsion Eisenfeld, Avi Karsenty

**Affiliations:** 1 Advanced Laboratory of Electro-Optics (ALEO), Department of Applied Physics/Electro-Optics Engineering, Jerusalem College of Technology (JCT), 9116001 Jerusalem, Israel; hyp7271@gmail.com (H.P.); tsioneisenfeld@gmail.com (T.E.); 2 Nanotechnology Center for Research and Education, Jerusalem College of Technology, 9116001 Jerusalem, Israel

**Keywords:** nano pixel, light emitting device (LED), nano display, modeling, physical simulations

## Abstract

A nano light emitting device (LED) has been developed and is presented. This new LED, entitled LENS (Light Emitting Nano-pixel Structure), is a new nano-pixel structure designed to enable high-resolution display. It serves as the building block of a more complex structure called LENA (Light Emitting Nano-pixel Array), dedicated to nano-display applications, such as augmented and virtual reality (AVR). Previously designed and studied with a platform for ray tracing optimization, a complementary simulations study was performed using the Comsol Multi-Physics Platform in order to check for opto-electronics performance and physical nanoscale investigations. In addition to the physical complementary analysis, several studies have focused on optimizations: optimal geometry for a pixel (cylindrical or conical shape), and wavelength adaptation (optical communication). In addition to numerical simulation results, an analytical model has been developed. This new device holds the potential to enhance the light efficiency for military, professional and consumer applications, and can serve as a game changer in the world of nano-displays with high resolution.

## 1. Introduction

While the domain of micro-light-emitting diodes (μ-LEDs) has become the focus of research for display applications [1], the world of nano-LEDs (n-LEDs) remains unexplored and several tentative approaches are still works in progress. In the last half-decade, one can observe several research directions focusing on nano-pixel technologies. While some teams are still focusing on more fundamental research on phase change materials between two layers [2], others are looking at more applied structures, like the structural and optical properties of InGaN/GaN quantum dots [3], nano-pixel matrix [4] and even nano-ring light-emitting diodes (NRLEDs) with different wall widths, fabricated by specialized nano-lithography technology [5]. Table 1 presents a summary of these studies.

When compared to the recent progress at the academy, looking at the advanced structures of nano-pixels and nano-display [6], several industrial technologies do not yet facilitate sub-micron pixels. Integral parts of these most well-known used technologies comprise the liquid crystal display (LCD) [7], the liquid crystal-on-silicon (LCOS) [8] combining liquid crystal (LC) materials and high-performance silicon complementary metal-oxide-semiconductor (CMOS) advantages, the organic light-emitting diode (OLED) micro display [9], and the digital light processing (DLP) [10]. Additional micro-scale technology includes the digital mirror device (DMD), which is a micro electro mechanical system (MEMS) device which was invented in 1987 [11]. Designed for projection usage, where the tilting mirror pixels reflect the light out of the projection lens, DMD usually generates high contrast images when compared to other display technologies, such as LCOS, LCD or OLED displays.

**Figure 1:**
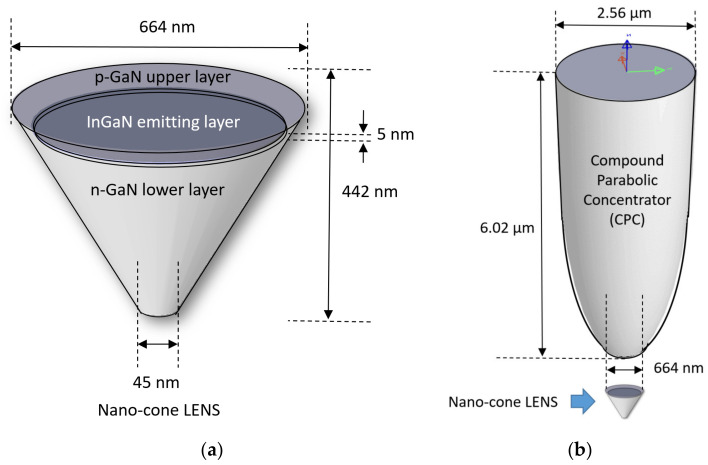
3D representation of the device structure. (**a**) Nano-cone light emitting nano-pixel structure (LENS); (**b**) Nano-cone LENS with compound parabolic concentrator (CPC).

LENS (light emitting nano-pixel structure), a new nano-metric LED, is designed, simulated, and modeled for feasibility analysis, with the challenge of combining high resolution and high brightness for display, essentially adapted for augmented reality (AR) and virtual reality. The device is made of two parts: The first one is a reflective nano-cone LED structure (Figure 1a) to reduce the total internal reflection effects (TIR) and enable improved light extraction efficiency. The second part—not considered in this article—is a compound parabolic concentrator (CPC), situated above the nano-LED (Figure 1b) to narrow the outgoing light angular distribution so most of the light is “collected” by an imaging system. Since pixel resolution is critical to offer advanced applications, an extensive feasibility study was performed. Pixel resolution is critical, in order to offer a valuable user-experience for military, industry and consumer applications.

In order to be appropriate for the above applications, a good display requires an equilibrium between high resolution, small dimensions and good color purity. For example, a monochromatic green nano-LED is usually based on InGaN/GaN materials (Table 1), since a direct GaN-based green LED emits a narrower spectrum than the phosphor converted green LED [12]. Two additional important parameters are display brightness [13] and power efficiency, since they allow—or do not allow—outdoor applications as well as a long battery life. Another challenge to overcome is thermal dissipation [14] while increasing the light efficiency and reducing the whole display working temperature.

To face the above challenges, concerns, and considerations—in particular the power efficiency issue—LENS combines two complementary parts. The first one is a reflective nano-cone LED structure. Its role is to mainly reduce the total internal reflection effects (TIR), and of course, to enable improved light efficiency. This article focuses on this part. The second part, placed above the nano-cone LED, is a compound parabolic concentrator (CPC). Its role is to narrow the outgoing light angular distribution in such a way that most of the light is “accepted” by an imaging system. When compared to a conventional flat shape LED, it was previously demonstrated that when combined, the cone nano-LED and the CPC parts improve the power efficiency by around 3800%. Initial publications of such super-high intensity nano-emitting (SHINE) pixels [15] and LENS have recently been presented [16], when the analysis is based on the ray tracing method. In the current article, we complete the previous ray tracing analyses by adding physical studies of the device’s behavior when varying physical parameters such as dimensions, geometry, wavelength (RGB), carriers’ concentration, efficiency and more. Three important planes are analyzed: The embedded emitting layer (x-y plane), the surface plane (x-y plane), and the energy levels plane along the z-axis (x-z plane). Going forward, we enlarged the study from green monochromatic to RGB.

**Table 1:** Comparative table presenting existing nano-pixel technologies and specifications.

Institute	Pixel Size	Technology	Emission	Material
Oxford University [2]	300 nm × 300 nm	Phase change material Amorphous to crystalline state	Non-emitting pixel Not colored. Require backlight illumination	Ge-Sb-Te + Indium Tin Oxide (ITO) electrode
Mc Gill and Mc Master Universities [3]	500 nm × 1000 nm	Dot in nanowire light emitting diode Varying nanowire diameter modulates wavelength emission	Self-emitting pixel RGB	InGaN/GaN
University of Illinois [4]	640 nm × 640 nm	Organic LED (OLED) Hierarchical multi-color nano-pixel matrices	Self-emitting pixel Multicolor	Ligand Polymer + Layer of Eu and Tb ions.
National Chiao Tung University [17]	800 nm diameter	Tunable wavelength InGaN/GaN Nano-ring LEDs via Nano-sphere lithography	Self-emitting pixel RGB	InGaN/GaN
ALEO [18] at JCT [15,16]	664 nm diameter	Sub-micron dimension Conical advanced shape Nano-LED	Self-emitting pixel Monochromatic RGB option	p-GaN/InGaN/n-GaN

## 2. Device Concept and Structure

### 2.1. Electro-Luminescence and LEDs

In a direct band gap semiconductor submitted to an electrical excitation, electrons and holes can recombine to produce a spontaneous light emission called electro-luminescence. In this physical process, an electron from the conduction band can relax and recombine with a hole into the valence band allowing the emission of a photon sharing an energy value matching the energy gap of the semiconductor. An electron–hole pair is created by introducing impurities into the semi-conductor to obtain p-type or n-type materials and bringing them together in order to make a p-n junction. This is, for example, how light-emitting diodes (LED) work [19]. An LED is a semi-conductor p-n junction, which emits light when under the influence of an electric field. Free to move in the semiconductor—as they are charge carriers—electrons cross the n-region to recombine with the holes of the p-region in what is called a “forward-biased” p-n junction. Two energy bands describe the range of energies in the electronic band structure of a solid state. The conduction band is populated with free electrons, which can move into the semiconductor, while the valence band is populated with holes. As a result, holes are located at a lower energy level than the conduction band electrons. At thermal equilibrium, the product of the electron and hole concentration is a constant. Under forward bias, a significant part of the net electric current of free carriers is dissipated in the form of electrons and holes recombination. The result of this is the creation of a radiative emission with photons (light) for a direct band gap semiconductor, or a non-radiative transition with phonons for an indirect band gap. In materials such as silicon, electrons and holes recombine resulting in a non-radiative transition producing no optical emissions because of their indirect gap band energy. Today, LEDs use different materials with direct band gaps, such as gallium arsenide, in order to enable emitting light devices in a large range of colors. Common LEDs are made of an n-type substrate and of an electrode on the p-type layer. LEDs use different substrates, such as silicon carbide (SiC) and gallium nitride (GaN). In spite of the fact that it is not a semiconductor, sapphire is also used as a transparent substrate. Several important studies using InGaN/GaN layers and related to new types of micro and nano LEDs were published in the last decade regarding several fabrication techniques and feasibility proof of concept. For instance, nano-rods fabrication was studied while focusing on wet etching techniques, and comparison between planar LEDs’ and nano-rods LEDs’ photo-luminescence (PL) [20]. Nano-wires InGaN/GaN light emitting diodes, based on vertically standing single nano-wires and nano-wire arrays were fabricated and extensively characterized and electro-luminescence (EL) was studied [21]. More recently, micro-LEDs’ electro-luminescence was also reported [22]. In the domain of the simulation, several numerical analyses were performed using Comsol Multi-Physics Software Package, as we also propose here. Among others, numerical simulations of the electric current and temperature distribution in GaN-based LED chips were performed. Such information can be used in order to improve the device design and optimization of the LED devices [23]. More investigations have focused on the LED’s efficiency [24]. Recently, InGaN LEDs were simulated for green and red wavelengths [25].

### 2.2. Design Considerations from Simple LED to Nano-Pixel

Our intention is to develop a self-emitting nano pixel, suitable for head mounted displays. There are several important parameters and added values that must be taken into account for this new design. The first one is the pixel size in order to reach higher image resolution and field of view and/or to shrink the size of the overall head mounted display’s optics. Improved nano-LED light extraction with a reflective conical structure, and a pixel with a narrowed angular distribution outgoing light to increase the image brightness and power efficacy are also required to allow for outdoor applications with longer battery life. As part of the specifications, the monochromatic pixels’ array will reduce the nano-display size by two thirds when compared to full RGB’s. For this purpose, we developed a nano pixel of 664 nm size without any condenser module. It is also important to obtain a self-emitting light type nano pixel: since the nano-pixel is based on a LED, it can itself emit light without an external light source, unlike LCD screens that require a back-light [26]. When compared to LCD, this will allow us to significantly reduce the thickness of the nano-display and of a potential imaging system. To do so, it will emit light when under electrical voltages using the phenomenon of electroluminescence by electron–hole recombination by direct gap [27]. The desired light emission wavelength is 525 nm, since green radiation is the most sensitive color for the human eye. In military applications, for example for pilot helmet mounted displays, a green image is common and suitable for day and night vision [28]. A monochromatic pixel array will allow us to reduce the pixel spacing, increasing the pixel density. Regarding the light beam angular distribution control, an optical condenser module, such as a compound parabolic concentrator [16] is considered to control the angular distribution of the light beam outgoing the pixel. Indeed, the numerical aperture is different depending on the system. Finally, we look at the efficiency. For a better efficiency, the pixel’s Numerical Aperture (NA) should best match the optical system’s NA, otherwise light will be lost without being “accepted by the system”. The advantages of LENS when compared to regular LEDs are presented alongside the numerical analyses. Device’s structure specifications, such as thickness, size, and doping level are presented in Table 2.

## 3. Methods

### 3.1. Numerical Preliminary Analysis—Ray Tracing Oriented Software at Micro Range

Monte-Carlo ray tracing (MCRT), also called Monte-Carlo path tracing (MRPT) is a computer-based graphics method dealing with the geometric approximation approach of the light propagation in a system or in a component. As largely explained [29], this method is based on the statistically predictable behavior of entities, called rays, which describe the paths followed by energy bundles as they are emitted, reflected, scattered, refracted, diffracted and ultimately absorbed. For macro and micro-systems, when reducing the dimensions of the investigated elements to the nanoscale level—below the light wavelength scale—such approximations become useless. While Maxwell equations are still capable of simulating light behavior in the nanoscale range, additional mechanisms and dependences of electrical parameters must be considered as well. This means that the analysis of a diode junction, for example, cannot be fully simulated when using only a ray tracing software. Analyses like a current–voltage curve, energy diagrams, active layer spectrum and other aspects of an LED cannot be obtained. Since the ray tracing approach ignores the opto-electronics behaviors of the material which is currently investigated, it becomes necessary to use additional physical software in order to complete the design of a nano-LED.

This means that the optical ray tracing software previously used to build the LENS device [15,16] is unable to predict the light behavior in sub-wavelength apertures, since this is not in its scope. Smaller and smaller pixels are of obvious interest and physical software contributes to a better understanding of sub-wavelength pixel efficiency and far field distribution. To finish, an array of pixels with a dedicated addressing scheme represents the road leading the way to the ultimate new generation of ultra-high efficiency and ultra-high-resolution displays.

### 3.2. Numerical Complementary Analysis—Physical Parameters Oriented Software at Nano Range

As mentioned above, complementary investigation of how light is extracted from the nano-LED pixel is required. When the minimum feature size of a light source is of the same order as that of the wavelength of light, a rigorous electromagnetic analysis is needed for calculating the light extraction efficiency of photonic crystal LEDs [30,31,32,33]. Classical radiometry and common Monte-Carlo ray tracing cannot perform such investigation [34,35]. Previous presented results of the device are mainly dedicated to the study of the rays optimal tracing [16] using LightTools software platform [36] and are also limited to micrometric scales. While choosing Comsol Multi-Physics Software Package [37], complementary investigations were oriented to physical and opto-electronics behaviors of the device, as presented below in next series of results. The numerical solution relies on the finite element method (FEM) [38], in which the geometry studied is divided into a finite element mesh as shown below [39]. Thus, instead of trying to solve a non-linear problem on the entire geometry, an approximate solution is sought in each element. If this element is considerably small, as is the case in our study, the physical problem is assumed to vary linearly. 

### 3.3. Analytical Analysis—Mathematical Review

In order to complete the picture, it was important to double-check the numerical results with an additional analytical approach, while calculating several parameters, like structures’ volume, carriers’ concentration, efficiency and more, using the relevant equations. This is why several analytical checks were performed in part of the paragraphs. Such results demonstrated the perfect match between the simulations and the calculations.

## 4. Numerical Results—Cylindrical vs. Conical Shape

The light emitting nano-pixel structure (LENS) is an opto-electronic device, coupling both electrical and optical behaviors in one complex structure. Among others, several behaviors were checked, such as carriers’ concentration, energy diagrams, electrical potential, emission rates and spectra, current-voltage (I-V) functionality and efficiency curves.

### 4.1. Structure, Layers and Geometry

**Figure 2:**
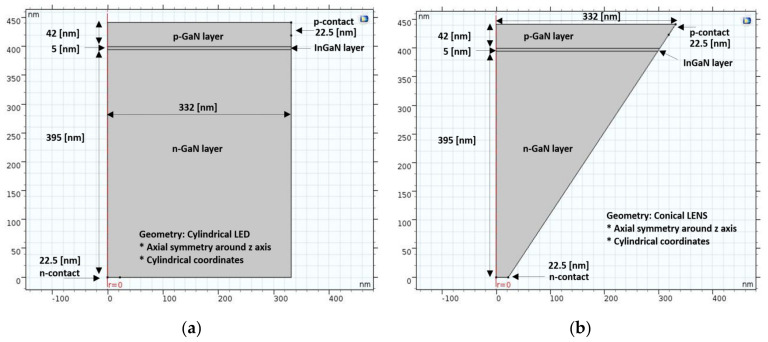
2D representation of the device structure, with an axial symmetry around the z-axis, and using cylindrical co-ordinates. (**a**) Cylindrical shape (regular device); (**b**) conical shape (LENS device).

Optimal shape and geometry influences were partially analyzed in the past in specific case studies, for example, while comparing three types of LED chips using different geometries of top surface on GaN p-n junction structures, such as domes [40] or triangular quantum barriers [41]. In our case, using multi-physics oriented software was also an opportunity to check for shape and geometry optimizations. Therefore, throughout this study, two basic shapes of pixels were checked and compared: cylinders (regular LED) vs. cones (enhanced LENS). The aim was to assure that the original conical shape of the LENS device shares advantages when compared to the regular cylindrical geometry. The first milestone in this systematic study was to design the two structures in parallel, as presented in Figure 2. A longer description of the structures, serving as a support to extensive simulations work, is presented below. In both the geometries, the device is made of the same three layers, sharing different roles: The p-GaN upper layer (t_em_ = 42 nm) is the surface plane, the InGaN middle layer (t_InGaN_ = 5 nm) is the emitting plane, and the N-GaN lower layer (t_sub_ = 395 nm) is the substrate. The upper diameter is equal in both geometries (D_top_ = 664 nm), while the lower diameter is reduced to D_base_ = 45 nm, in the case of conical frustum shape (LENS device). All geometrical dimensions are compared and presented in Table 2. Regarding the emitting layer, In(x)Ga(1-x)N is a non-stoichiometric compound and its band gap can be tailored by adjusting the stoichiometric parameter x, defined as the molar fraction of indium in the compound. Since the simulation software does not include such a dependence, we defined an artificial InGaN, and parametrized the band gap energy to match the desired emission wavelength. As for the layers, one can also distinguish different axes of direction: the horizontal x-axis presents the radius (*r*) of the surface plane, while the vertical z-axis presents the direction of the quantum wells.

### 4.2. Mesh Density and Accuracy

**Figure 3:**
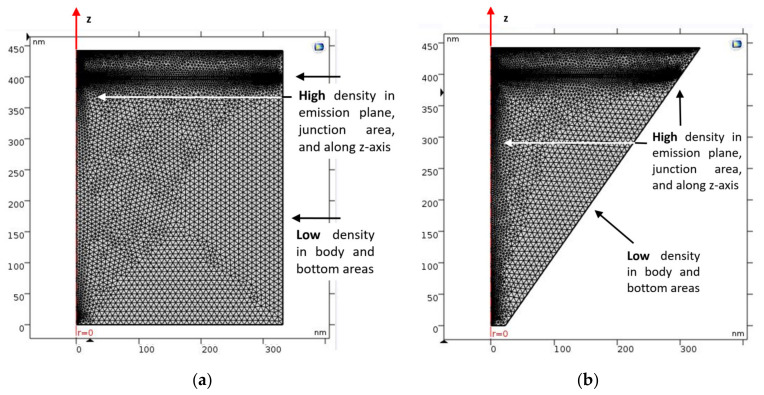
2D representation of the device mesh density distribution, with an axial symmetry around the z-axis and using cylindrical co-ordinates. (**a**) Cylindrical shape (regular device); (**b**) conical shape (LENS device).

While the devices are presented in Figure 2a,b, their corresponding meshes are shown in Figure 3a,b. The geometries of the device are discretized in squares to achieve the most accurate results for their block symmetry. The discretization is denser in some parts—around the emitting plane and on the surface plate—in order to obtain results that are more accurate. The remaining geometry is discretized in a tetrahedron, which is the default mesh. The mesh used in the 2D simulation is the default mesh and is different from the 3D one. The chosen element size option was “extra fine”.

As part of this geometry comparison study, it was important to summarize in one place (Table 2) all the parameters which were used for the 2D/3D simulations. One will pay attention to the point that all the defined parameters are similar for both the LED and LENS structure, except the geometry dimensions.

### 4.3. Electron and Hole Concentrations and Distribution (x-axis)

**Figure 4:**
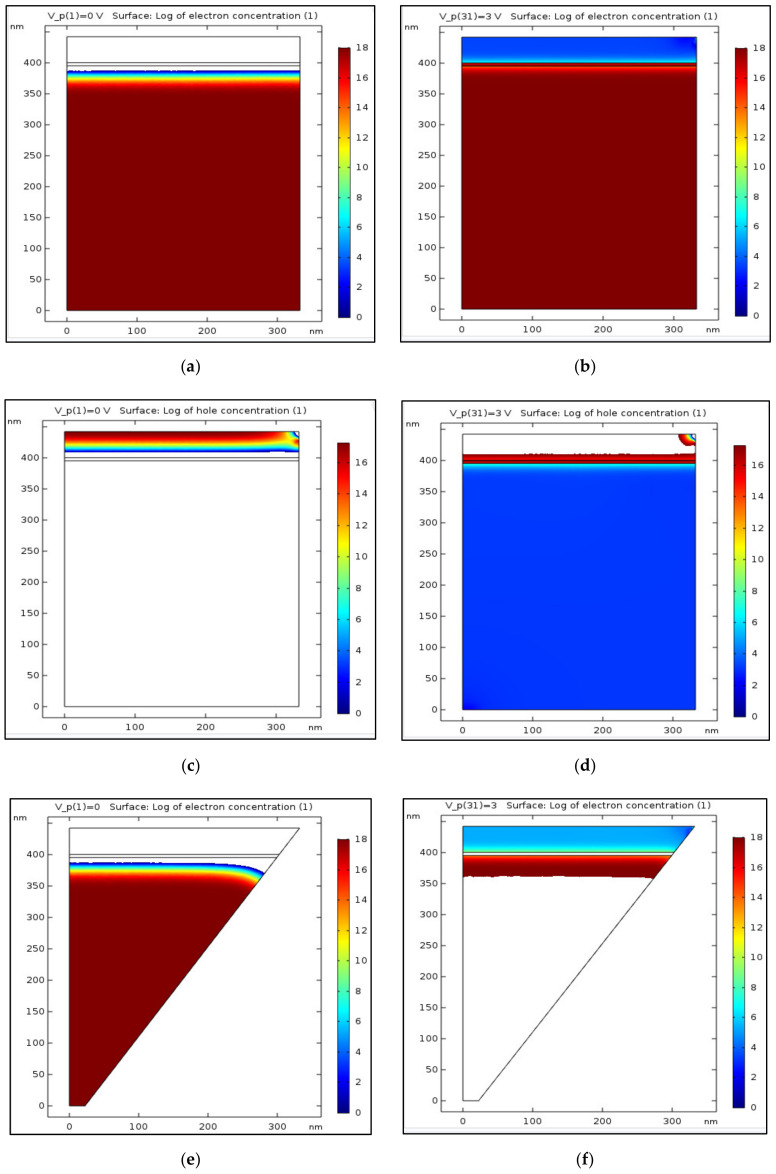

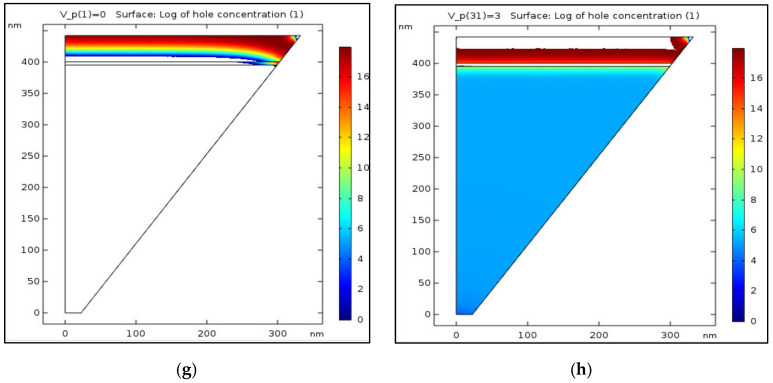
2D simulations of electrons and holes concentration in cylindrical shape (LED) at equilibrium (no voltage bias) and at 3 V. Color Log distribution scale in cm^−3^. T = 300 K, λ = 450 nm, E_gap_ = 2.75 eV. Two-dimensional LED: (**a**) electrons, V = 0 V; (**b**) electrons, V = 3 V; (**c**) holes, V = 0 V; (**d**) holes, V = 3 V. Two-dimensional LENS: (**e**) electrons, V = 0 V; (**f**) electrons, V = 3 V; (**g**) holes, V = 0 V; (**h**) holes, V = 3 V.

**Figure 5:**
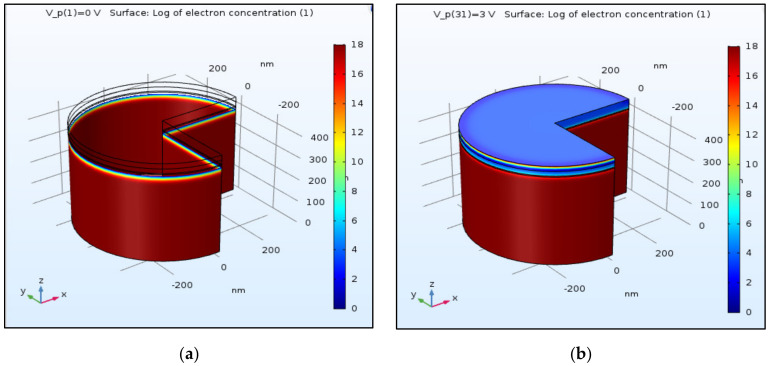

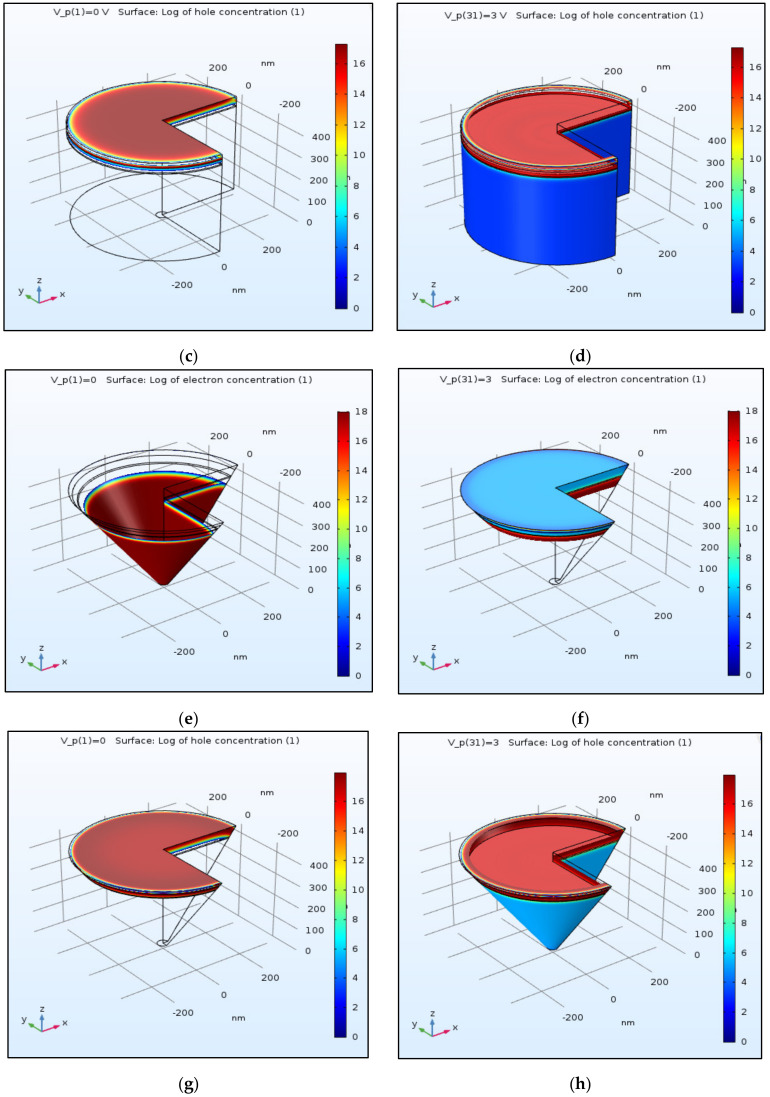
3D simulations of electrons and holes concentration in conical shape (LENS) at equilibrium (no voltage bias) and at 3 V. Color Log distribution scale in cm^−3^. T = 300 K, λ = 450 nm, E_gap_ = 2.75 eV. 3D LED: (**a**) electrons, V = 0 V; (**b**) electrons, V = 3 V; (**c**) holes, V = 0 V; (**d**) holes, V = 3 V. Three-dimensional LENS: (**e**) electrons, V = 0 V; (**f**) electrons, V = 3 V; (**g**) holes, V = 0 V; (**h**) holes, V = 3 V.

After focusing on the structure and on the mesh, it is now important to investigate the carriers’ behavior at different planes. As described above, there are two important planes in this device: the “emitting plane” and the “surface plane”. The emitting plane is the InGaN layer, which is embedded between two other layers: the upper surface p-GaN layer and the lower n-GaN substrate. It is interesting to understand how the carriers—electrons and holes—are distributed along this plane, from the center to its periphery. As shown below, this distribution was, respectively, simulated at equilibrium V = 0 and at V = 3 V. This value corresponds to a forward bias for which the device should operate in 2D and 3D modes, for both the LED (Figure 4a–h) and LENS (Figure 5a–h). If the red color emphasizes the regions sharing a high density of the relevant carriers, the blue one emphasizes the low-density zones. Doping concentration values are defined as 10^18^ cm^−3^ for both the p and n regions, and for both the geometries (cylindrical and conical). At room temperature, the semiconductor should be extrinsic so the electron and hole concentrations equal the n and p-dopant concentration, respectively. One can observe that when compared to the cylindrical shape, the cone’s right edge exhibits a curved gradient of the carriers’ distribution, which may be due to the non-zero gradient of the dopant distribution if the z-direction for the conical device. The smaller gradient curvature observed at the right and left edges of the cylindrical structure may be due to the pinning of the Fermi level, related to recombination of the carriers with surfaces states or to the depletion layer at the edge, due a probable definition of the boundaries as a rectifying instead of pure ohmic contacts. The units are presented in cm^−3^ and the low log value of the concentration is due to the very low intrinsic concentration of the GaN layers 1.9 × 10^−10^ cm^−3^ at 300 K since its large band gap (3.43 eV). The intrinsic InGaN layer may be depleted at equilibrium. In order to present a color concentration scale starting from zero (i.e., without negative values), in the electron zones the inexistent holes will appear in white color, and vice-versa.

One can observe that the carriers’ density under an applied voltage is higher in the LENS conical device than in the cylindrical LED. One can assume that this phenomenon, probably caused by the different geometry, enables a better recombination.

### 4.4. I-V Curves

**Figure 6:**
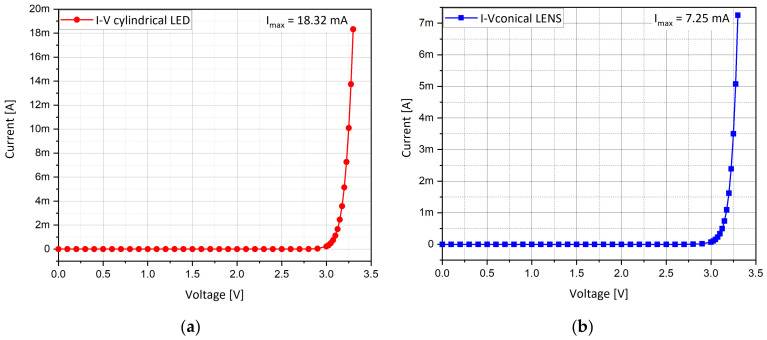
(**a**) I-V curve of cylindrical LED; (**b**) I-V curve of conical LENS. V = 3.3 V, λ = 450 nm, E_gap_ = 2.75 eV.

An additional sanity check is related to the I-V curves behavior. At the end, when developing a new device and presenting its specifications, some curves become representative of its functionality. In our case, we can identify the I-V curve of a diode (Figure 6) as significant, where V is the forward bias applied to the top of the p-GaN layer relative to the bottom of the n-GaN layer. The main difference is the measured current intensity. For a bias of 3.3 V, the calculated current for the conical LENS device is found to be about a third of the calculated current for the cylindrical device.

The current ratio is related to the volume ratio between the structures. The lower maximal value observed in the LENS I-V curve can be explained by the fact that there are less charge carriers, as a consequence of the geometry shape. Based on the parameters presented in Table 2, one can now calculate the ratio of the volumes and compare it to the ratio of the currents.

The volume of a cylinder (LED device) is given by:

(1) $$V_{cylinder}=\pi r^{2}\;\times \;h$$

where:

- *r* = is the radius of the cylinder’s circle,
- *h* is the height of the cylinder.

In the case of our LED, while replacing the values of the structure, we obtain:

(2) $$V_{cylinder}=\pi \;\times \;{\left(332\right)}^{2}\;\times \;442=0.15\;\mu m^{3}$$

The volume of a conical frustum shape (LENS device) is given by:

(3) $$V_{conical\;frustrum}=\frac{1}{3}\;\pi h\left(r^{2}+rR+R^{2}\right)$$

where:

- *r* = is the radius of the cone’s small circle,
- *R* = is the radius of the cone’s large circle,
- *h* is the height of the cone.

In the case of our LENS, while replacing the values of the structure, we obtain:

(4) $$V_{conical\;frustrum}=\frac{1}{3}\pi \;\times \;442\left[22.5^{2}+\left(22.5\right)\times \left(332\right)+332^{2}\right]=0.54\;\mu m^{3}$$

While looking for the ratio of the volumes, we obtain:

(5) $$R_{volumes}=\frac{V_{conical\;frustrum}}{V_{cylinder}}≅\;0.3$$

It appears that both the ratios, $R_{volumes}$ and $R_{currents}$, are equal, so one can explain the consistency of the electrical results as a function of the geometrical shape.

(6) $$R_{currents}=\;\frac{I_{conical\;frustrum}}{I_{cylinder}}≅\;0.3$$

(7) $$R_{volumes}≅R_{currents}$$

### 4.5. Carriers Concentration Along x-axis and z-axis

**Figure 7:**
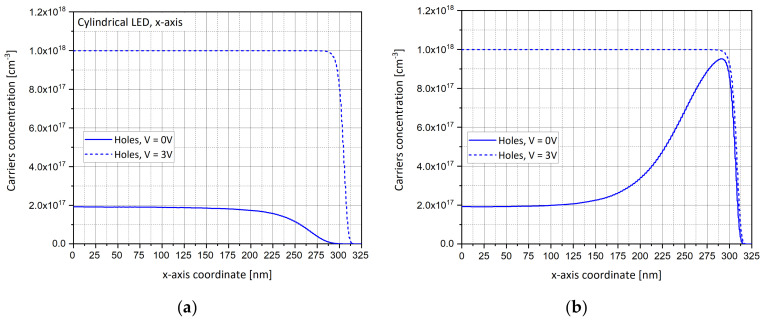

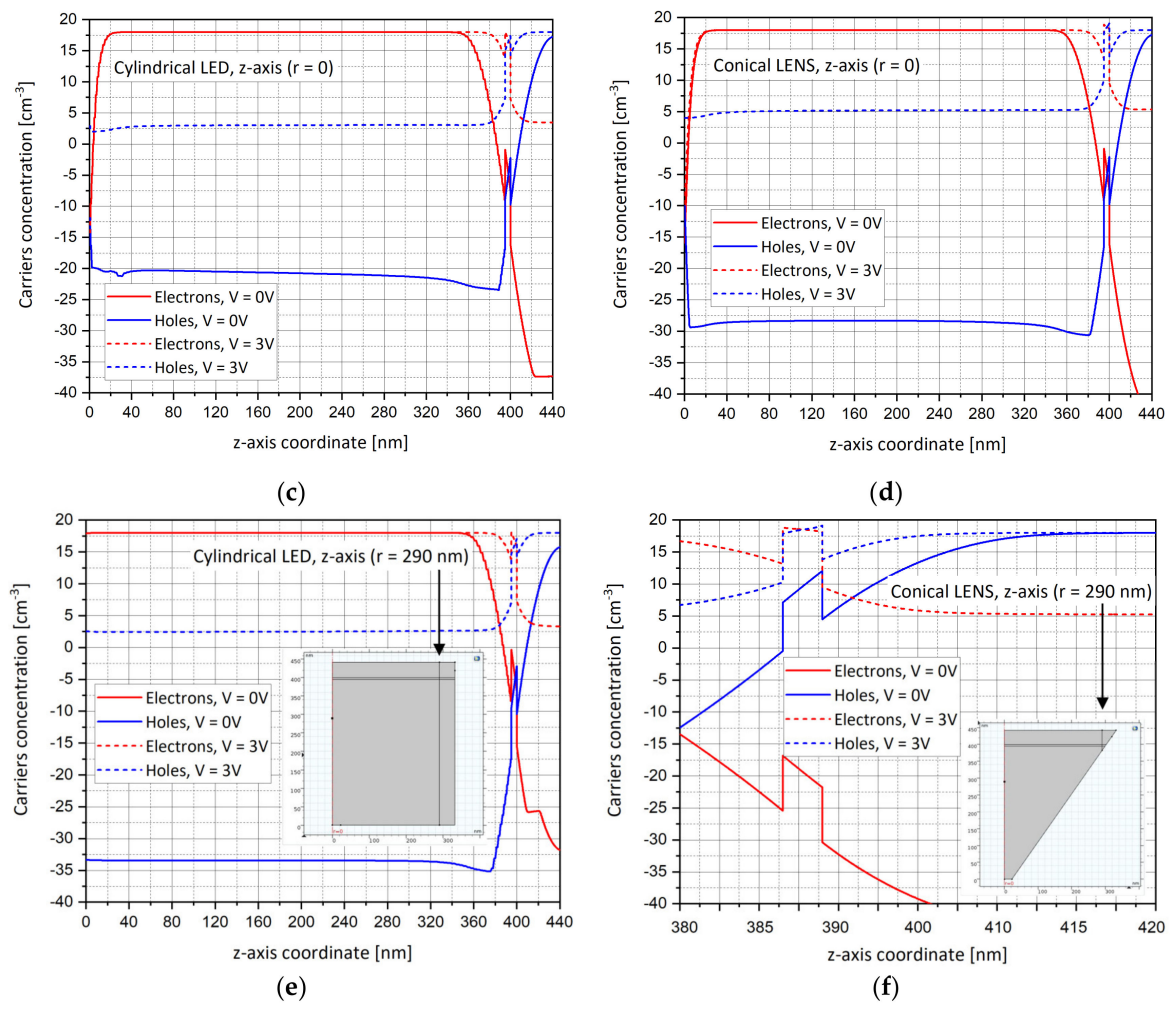
Simulations of carriers’ concentration for zero bias (0 V) and forward bias (3 V). (**a**) Holes’ concentration for cylindrical shape (LED) along x-axis (z = 442 nm, surface plane), There is a depletion of majority carries (holes) near the edge for the cylindrical device; (**b**) Holes’ concentration for conical shape (LENS) along x-axis (z = 442 nm, surface plane). There is an accumulation of holes near the edge for the conical device. (**c**) Carriers’ concentration for cylindrical shape (LED) shape along z-axis (r = 0); (**d**) Carriers’ concentration for cylindrical shape (LENS) shape along z-axis (r = 0). At r = 0, both LED and LENS present similar distributions. (**e**) Carriers’ concentration for cylindrical shape (LED) shape along z-axis (r = 290 nm); (**f**) Carriers’ concentration for cylindrical shape (LENS) shape along z-axis (r = 290 nm).

Since the surface layer is p-type (p-GaN), we are interested in looking after the profile of the holes’ distribution and values along the radius. This is why in addition to the above “visual” 2D and 3D distributions, holes’ concentration profiles are presented below, showing the respective decreasing (Figure 7a) and increasing (Figure 7b) extremities of the curves along the x-axis. Similar observations with carriers’ concentration along the z-axis (r = 0) are presented in Figure 7c,d. The concentrations are presented as a function of the radius coordinate (r) in nanometers along the x-axis or simply in depth along the z-axis and are expressed in per units of volume per cm^3^.

One can observe that, for the conical shape, the curve remains almost linear in both the graphs, until a radius value of 150 nm and then the distributions slightly decrease/increase, respectively, before changing their slope to an inverse strong behavior at the cone’s extremities. For the LED there is a depletion of majority carries (holes) near the edge for the cylindrical device, while for the LENS there is an accumulation of holes near the edge for the conical device. A possible explanation for this phenomenon is the fact that the holes’ concentration in the layer above the depletion one is influenced by two factors: the depletion zone, generated because of the junction, and the positive contact point. Additionally, a non-zero (linear) gradient of the doping in the z direction can also cause the depletion layer. One can assume that the boundary at the oblique edge is defined as rectifying instead of ohmic. While the depletion layer pushes the holes up towards the surface, the positive contact point pushes them inside the LED. In a cylindrical LED, the more we progress along the radius towards the extremity, the more the positive contact point is dominant and the holes’ concentration decreases. In the conical LENS structure, the slope of the side wall influences the shape of the depletion layer, so more holes succeed to overcome the positive influence of the contact point, until they reach the situation that the rejection force becomes dominant when compared to the depletion layer influence.

One can observe (Figure 7a,b) that the holes’ concentration quickly increases when approaching the right edge of the conical LENS structure. In fact, in the LENS the holes’ concentration is pinned close to the right edge at a maximum level almost equal to the p-type doping concentration of 10^24^ m^−3^ (or 10^18^ cm^−3^). Near r = 0, the hole concentration is almost 2.10^23^ m^−3^ (2.10^17^ cm^−3^). In the LED, the concentration of the holes decreases steadily from 2.10^23^ m^−3^ (2.10^17^ cm^−3^) to zero at the right edge of the structure. 

There are five times more holes in the conical structure than for the cylinder. We can therefore expect the conical shape to be more efficient. More holes mean more available electron–hole recombination pairs and as a consequence, more emitted photons. This means that the extremity of the LENS structure will be brighter than its center. On the other hand, regarding the LED cylinder, the brightness will drop along its radius. This behavior (decreasing holes in the LED and increasing in the LENS), can be explained with the enhanced gradient of the potential at the edge of the LENS. The enhancement of the concentration of the holes is seen at the right edge of the InGaAs region for the LENS device (also shown in Figure 4g).

## 5. Numerical Results—RGB Wavelength Optimization

Based on the above results, and after reaching the conclusions that the LENS conical shape structure shares many more advantages and better results, when compared to the LED cylindrical shape structure, it was important to check if these results are correct not only for the wavelength λ = 450 nm (blue), but if they are also consistent for other wavelengths. This is the reason why two additional wavelengths were checked for this new analysis: λ = 525 nm (green) and λ = 650 nm (red). The choice of these two values can be explained by the need to make an RGB coding system to generate all the color spectrum. 

**Figure 8:**
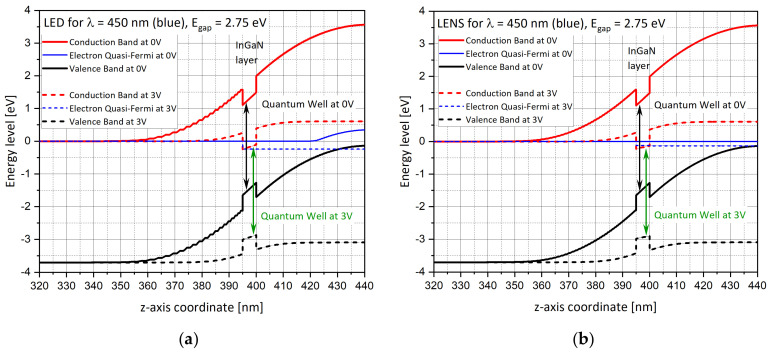

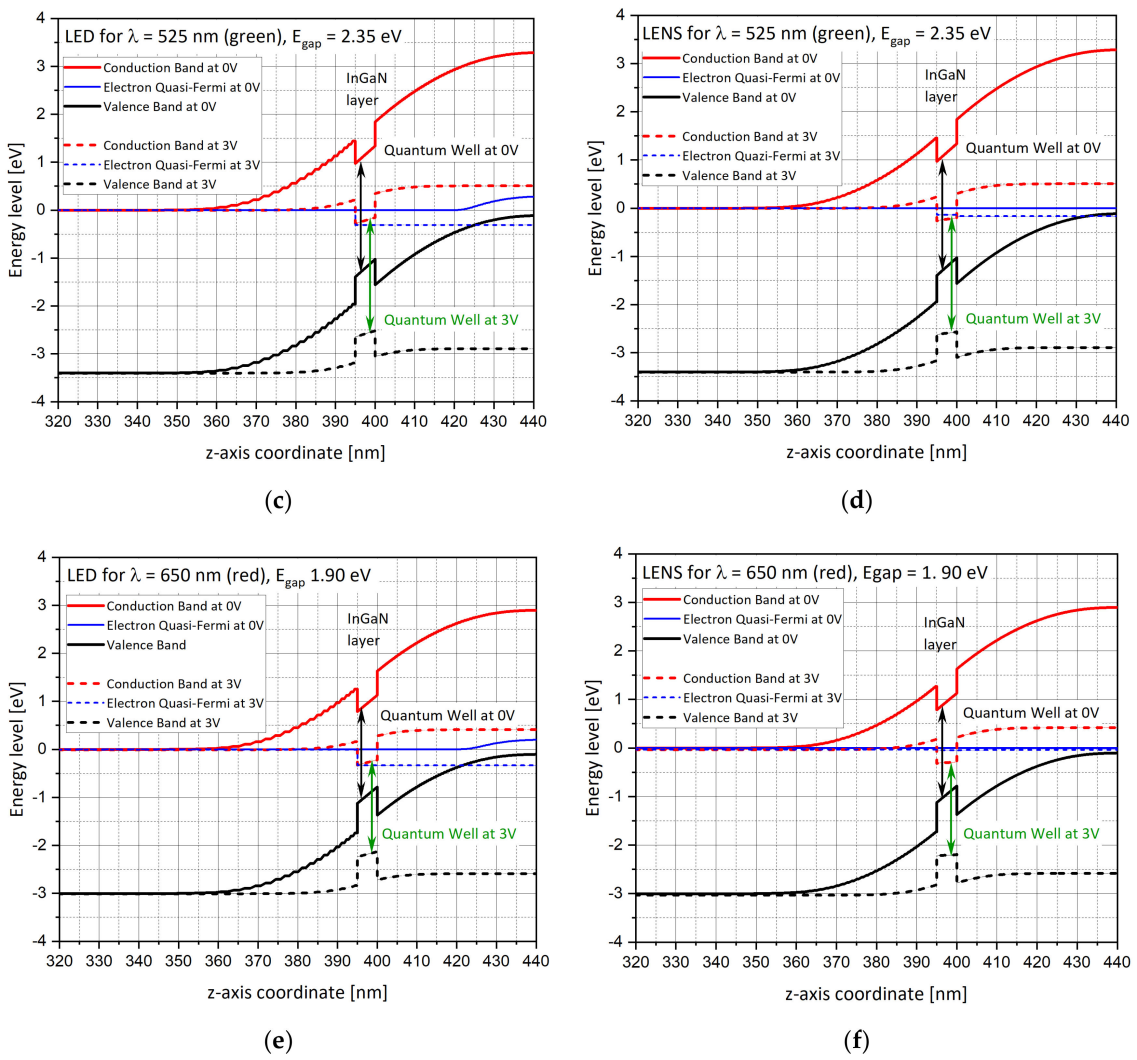
Energy diagrams as a function of the applied voltages, for V_p_ = 0 V and for V_p_ = 3 V, along the z axis (r = 0), and showing the PIN junction. (**a**) LED, λ = 450 nm; (**b**) LENS, λ = 450 nm; (**c**) LED, λ = 525 nm; (**d**) LENS, λ = 525 nm; (**e**) LED, λ = 650 nm; (**f**) LENS, λ = 650 nm.

**Figure 9:**
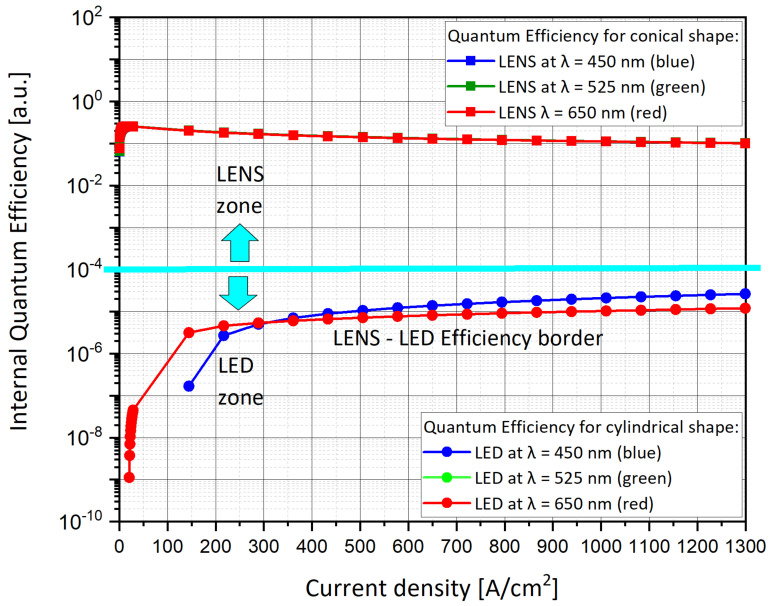
Nano-pixels specification graphs. Efficiency curves for cylindrical device (LED) vs. efficiency curves for conical device (LENS). The blue and green LENS curves are overlapping, while the blue and green LED curves are also overlapping.

**Figure 10:**
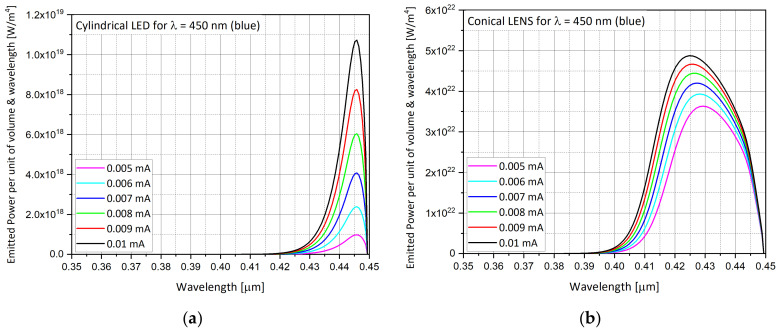

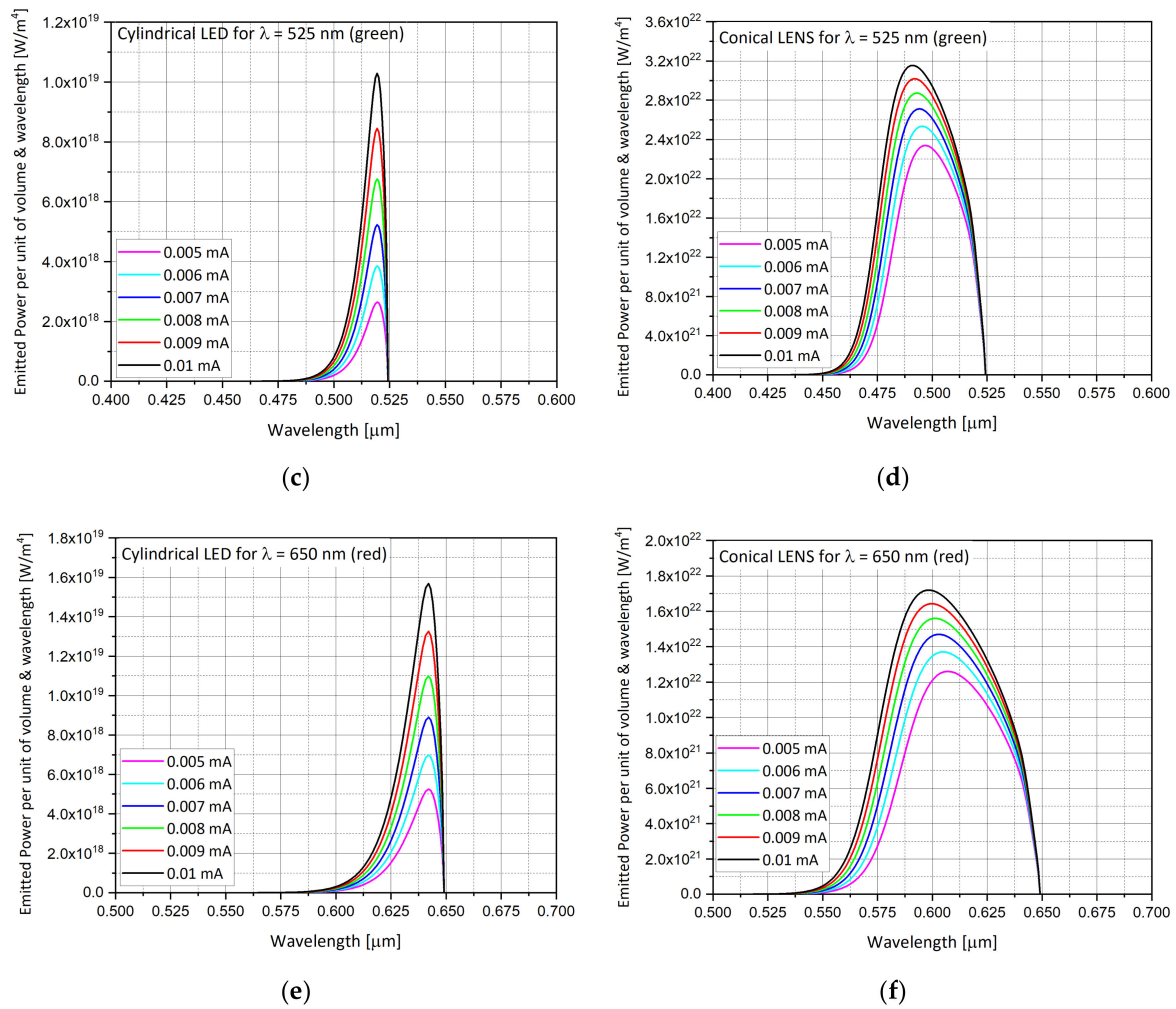
Emission spectra obtained from the InGaN layer. (**a**) LED, λ = 450 nm (blue); (**b**) LENS, λ = 450 nm (blue); (**c**) LED, λ = 525 nm (green); (**d**) LENS, λ = 525 nm (green); (**e**) LED, λ = 650 nm (red); (**f**) LENS, λ = 650 nm (red).

**Figure 11:**
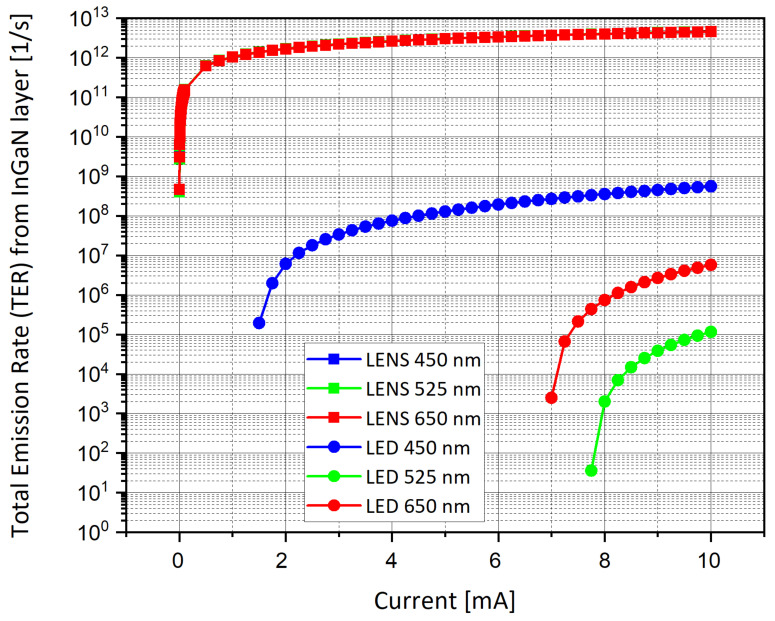
Total emission rate (TER) from the InGaN layer for several wavelengths for cylindrical and conical devices.

The next step was to choose which graphs to compare. In order to minimize the checks and to obtain quick and consistent answers, we decided to focus on several parameters as a function of the wavelength, and to compare them. The main analyses focused on the energy band diagrams (Figure 8a–f), the efficiency (Figure 9), the emission spectra from the embedded InGaN layer (Figure 10a–f), and the total emission rate (Figure 11).

### 5.1. Energy Band Diagrams (z-axis, r = 0)

After completing the first design phase by building the geometries of the cylindrical LED and conical LENS devices, and after the second phase for which it was necessary to adapt the optimal mesh to these structures, it became important to perform some sanity checks. The aim was to show that the simulation results are coherent with the expected physical behaviors. The first one is the calculation of the energy band diagrams for both of the studied shapes for selected bias values of 0 V and 3 V, as presented in Figure 8. The energy levels are calculated along the z-axis, for arc length *r* = 0 nm, crossing p-type GaN, InGaN and n-type GaN layers. If E_c_ is the conduction energy level, and E_v_ is the valence energy level, two additional levels are also presented: Ef_n_ and Ef_p_, which are the quasi Fermi energy levels of the electrons and the holes, respectively. E_fn_ is closed to E_v_ as expected of p-type material (doping of p-type GaN is 10^18^ cm^−3^) for both devices. The simulations are performed for RGB colors, comparing the LED (Figure 8a,c,e) to the LENS (Figure 8b,d,f) structures. Since the InGaN has a direct band gap, the band gap was selected to match the photon energy of a given emission wavelength [42]. The InGaN layer is an intrinsic material (n = p = ni) as the quasi Fermi levels of both electrons and holes lay in the middle of the InGaN bandgap at 0 V. The +3 V potential is applied to the top surface of the p-GaN layer (z = 442 nm), while the bottom surface of the n-GaN is grounded (z = 0). One can notice that, only in the LENS structure, an “inversion of population” occurs in the InGaN layer at 3 V bias since the electron quasi Fermi level is found higher than the conduction band level in the InGaN layer. The smaller the band gap is (or the longer the emission wavelength) the higher the electron quasi Fermi level and so the higher the electron concentration.

Additionally, the main difference occurring at 3 V between the LENS and the LED is that the E_fn_ is closer to the valence band, which is consistent with the previous results seen in Figure 7a,b where the concentration of the holes is higher for the LENS than for the LED at the right edge at 3 V.

Looking more deeply at the energy level diagrams above, for both the LED and LENS devices, and for zero bias (0 V) and forward bias (3 V) configurations, one can observe that the InGaN layer, which spans 0.395 ≤ z ≤ 0.400 μm, creates a potential well within both the conduction and valence bands. The potential barrier between the n-type and p-type sides of the device is reduced by the application of the forward bias. The conduction and valence bands are labeled E_c_ and E_v_, respectively, and the electron and hole quasi Fermi levels are labeled E_fn_ and E_fp_.

One can observe ripples in both the conduction and valence band curves in the above energy diagrams. The ripples appear only in the LED device curves (Figure 8a,c,e), when compared to the smooth curves in the LENS device. The source of these ripples is related to the accuracy of the mesh used in Comsol Multi-Physics. While for the LENS device, the finite elements’ density used for the mesh was very high, bigger mesh elements were used for the LED. The mesh choice is recognizable in the LED curves themselves. In fact, while zooming in on the LENS curves (Figure 8b,d,f), one can observe very small ripples as well. In the finite elements method (FEM), on which Comsol Multi-Physics software (SW) Package is based, the higher the number of mesh elements, the smoother the curve. It is similar to calculating an integral with small rectangles; the obtained curve’s accuracy will depend on the number and on the size of the used elements. The trade-off considerations are a longer simulation run time (days instead of hours) and bigger simulation files (tens of GB), etc. Another important note is related to Figure 8f. A priori, it may look strange that the quasi Fermi curve E_fn_ remains constant, even when changing the bias from 0 to 3 V. In fact, when looking at the values themselves, they are different, however, the difference is too small to be distinguished between the two bias curves.

As mentioned above, the InGaN emitting layer is an intrinsic material where:

(8) $$n=p=n_{i}$$

as the quasi Fermi levels of both electrons and holes lay in the middle of the InGaN bandgap at 0 V. This important value (n*_i_*) can be calculated according to [43] and as a function of the band gap:

(9) $$n_{i}^{2}=N_{C}N_{V}e^{-E_{g}/K_{B}T}$$

where:

- $N_{C}$ and $N_{V}$ are the effective densities of states in the conduction and valence bands;
- $E_{g}$ is the band gap;
- $K_{B}$ is Boltzmann’s constant;
- T is the lattice temperature.

### 5.2. Efficiency Curve vs. Current Density

Last but not least, is the study of the efficiency curve as a function of the current density. Efficiencies are defined differently based on the type of device. For example, for photodetectors based on semiconductors, one can distinguish two separated efficiencies: the internal quantum efficiency (IQE) and the external quantum efficiency (EQE) [44]. The IQE, also named *η_int_*, is defined as the ratio of the number of electron–hole (e–h) pairs or charge carriers generated to the number of photons absorbed, within the active layer(s) of the device, in our case InGaN emitting layer. It is also called the quantum yield and accounts for the recombination loss. Typically, for a superior quality material with low dislocation density and defects, IQE could be close to 100% if absorption due to free carriers is negligible. The EQE, also named *η_ext_*, is defined as the number of photo-generated charge carriers (coming out as photocurrent from the device) measured across the detector divided by the number of photons incident on the device. For any detector, the EQE is lower than the IQE primarily due to two factors. The first factor is related to the fact that not all incident light is absorbed in the active layer due to various losses such as Fresnel loss at the air/semiconductor interface, reflection at the metal electrodes, absorption within the non-active layers of the device, etc. The efficiency corresponding to these processes is clubbed into *η_t_*, transmission efficiency, the fraction of incident light which manages to reach and get absorbed in the active layer(s). The second factor is related to the fact that not all photo-generated e–h pairs manage to “come out” of the device as electric current. This is captured in the collection efficiency, *η_c_*. At the end, we obtain:

(10) $$\eta_{ext}=\eta_{int}\eta_{t}\eta_{c}$$

where:

- $\eta_{ext}$ is the external quantum efficiency;
- $\eta_{int}$ is the internal quantum efficiency;
- $\eta_{t}$ is the transmission efficiency;
- $\eta_{c}$ is the collection efficiency.

For the light emitting device (LEDs), the efficiency is defined differently [45], as per Equation (11):

(11) $$\eta_{q}=\frac{R_{r}}{R_{r}+R_{nr}}$$

where:

- $\eta_{q}$ is the internal quantum efficiency;
- $R_{r}$ is the rate of radiative recombination;
- $R_{nr}$ is the rate of non-radiative recombination.

As observed (Figure 9), the quantum efficiency of the conical LENS is much higher, by at least several orders of magnitude than the quantum efficiency of the cylindrical LED.

### 5.3. Emission Spectra from InGaN Layer

The emission spectra are presented for the RBG colors. The intensity units are presented as the emitted power per unit volume and energy. One can observe that for the conical shape (LENS device), the emission spectrum is larger (Figure 10b,f), and the peak of the intensity is always higher (Figure 10b,d,f), when compared to the cylindrical shape (regular LED), as presented in Figure 10b,d,f, respectively. This will affect the color purity of the LENS. The larger the spectrum the lower the color purity. Additionally, the peak intensity is not located at the same wavelength. The LENS peak intensity has a shorter wavelength. In reality this is less good when considering the photopic curve. The human eye is less sensitive to 430 nm (LENS peak) than to 445 nm (LED peak). A possible explanation of these differences may be the fact that more photons succeed in overcoming the full reflection phenomenon from the internal reflective walls of the LENS. When compared to the previous ray tracing analyses [15,16], where the reflective walls were dominant, here the reflection phenomenon has not yet been taken into account in this kind of simulation. Additionally, this may be due to the charge distribution and the energy band diagram profile. In Figure 10e,f, this time for the red color, the peak intensity wavelength is at the advantage of the LENS in regard to the photopic curve.

At the end, one can pay attention that there is no visible blue shift when increasing the intensity (Figure 10a,b). For each particular case, cylindrical LED or conical LENS and for each particular color, one can see that the spectrum remains unchanged but the emitted power is increased as a function of the current. For example, the cylindrical LED blue spectra range is always from 0.415 μm to 0.45 μm, independent of the current.

### 5.4. Total Emission Rate

**Figure 12:**
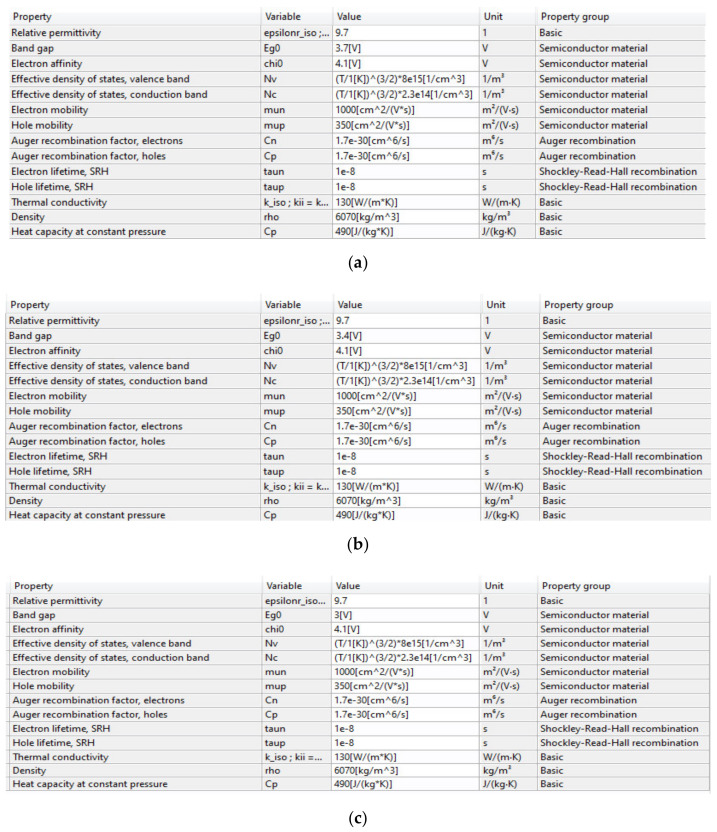

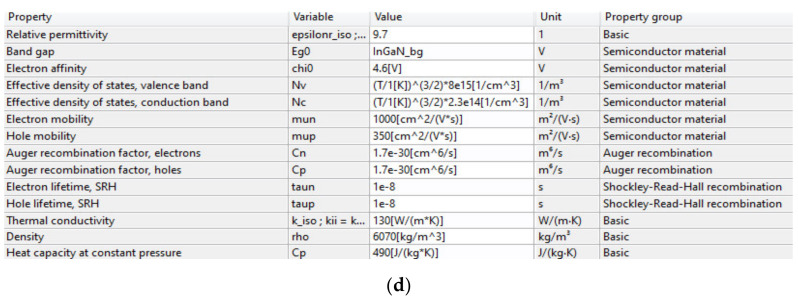
Summary of the physical parameters used in RBG study, using Comsol Multi-Physics Software Package. (**a**) GaN layer parameters for λ = 450 nm; (**b**) GaN layer parameters for λ = 525 nm; (**c**) GaN layer parameters for λ = 650 nm; (**d**) InGaN layer parameters.

The Total Emission Rate (TER) is a very important parameter and specification of such a device. Here again, the same conclusions obtained for the internal efficiency are reproducible in this case, as presented in Figure 11. One can pay attention that the curves are overlapping for the LENS. All the parameters used in the RGB simulations are summarized in Figure 12. As one can observe in this last figure, Comsol software enables us to set parameters as absolute values or as equations (more generic for replacing values), while the syntax is specific to the software definition. This makes the equations appear differently from a usual mathematical piece of writing. In our specific case, the equations for the effective densities of states for both valence and conduction bands were inserted as parameters and normalized to be expressed in m^−3^ units.

It is interesting to pay attention to the two families of curves presented in Figure 11 above. The LENS device presents better results than the LED one in several aspects. First, not only are all three RGB curves well matched in the conical LENS device, but they also present higher TER values, while the corresponding RGB curves are all separated and lower for the cylindrical LED. This means that the conical shape enables a higher emission rate. This TER is equivalent for any wavelength of the RGB standard. Second, the LENS device enables high values of TER even for a small current, while the LED structure requires higher current values to enable some light emission. The better results shown above for LENS do not consider the light reflections on the internal reflective walls, as previously published [16].

The internal quantum efficiency (IQE) and the total emission rate (TER) of the InGaN layer are directly linked. As seen in Figure 9, the LENS IQE is four orders of magnitude higher than the LED one. Furthermore, in Figure 11 one can see the similar improvement between the two structures. This can be can understood by mathematically expressing the TER as a function of the IQE.

(12) $$TER=\frac{P}{E}$$

where:

- P is the light power output;
- E is the photon energy.

Then, one can express the light power *P* as a function of the internal quantum efficiency:

(13) $$P=\frac{E}{T}\;\times \;\varphi$$

where:

- T is the emission time;
- φ is the quantity of emitted photon.

Since the quantity of emitted photons depends on the radiative emission rate, one can say that:

(14) $$P\;\sim E\;\times \;R_{r}$$

where:

- $R_{r}$ is the radiative recombination rate.

The radiative recombination rate can be expressed as a function of the internal quantum efficiency:

(15) $$R_{r}=\frac{-\eta_{q}\times R_{nr}}{\eta_{q}-1}$$

where:

- $\eta_{q}$ is the internal quantum efficiency;
- $R_{nr}$ is the non-radiative recombination rate.

Then we obtain:

(16) $$P\sim E\;\times \;\frac{-\eta_{q}\times R_{nr}}{\eta_{q}-1}$$

And:

(17) $$TER\sim \frac{E\;\times \;\frac{-\eta_{q}\times R_{nr}}{\eta_{q}-1}}{E}$$

Then:

(18) $$TER\sim \frac{\eta_{q}\times R_{nr}}{1-\eta_{q}}$$

This expression of the total emission rate is a positive value. One can see that when increasing the IQE, the TER is also increased in a similar manner.

## 6. Duality Applications

In its name, LENS—light emitting nano-pixel structure—includes a duality of roles which can be used for several applications. Firstly, as mentioned above, it is a new nano-pixel structure designed to enable high-resolution display, while it serves as the basic unit of a more complex structure called LENA (light emitting nano-pixel array), dedicated to nano-display applications, such as augmented and virtual reality (AVR). On the other hand, it is also a light emitting device, which can be integrated as a light source into a module, like an emitter-waveguide-receiver building block, to enable more complex functions. In this discussion, we will shortly review the diversity of applications for such a novel device, from high resolution display (HRD) to sensor functions.

### 6.1. LENS as a Pixel for Nano-Display

A priori, the super high brightness is only needed in augmented reality (AR), and not in virtual reality (VR) applications. Moreover, see-through AR optical designs have an inherently low efficiency, plus the output must be very bright to compete with high ambient light conditions such as daylight. Non-see-through VR designs are not only more efficient, but they can be much dimmer since the image produced does not compete with ambient light. Indeed, the AR optics domain requires very bright displays. See-through optical engines are generally based on waveguides that convey the image to the user’s eye. Because of the “transparent” see-through coupling-out and of the poor waveguide efficiency (from diffractive optics for example), more and more brightness is needed to compete with day light (several thousand of nits). For example, some recent systems use RGB laser diodes to compensate for the waveguide’s lack of efficiency. Even in this way, the system’s brightness is limited to only 500 nits. This is not enough for outdoor applications. Concerning the VR headsets, it is true that brightness is not an issue at all. Instead, battery time life could be improved by using more efficient displays. Nano LED displays have the potential to be more efficient than the ones currently available, such as OLEDs. Thus, our investigation is clearly of interest for VR optics.

### 6.2. LENS as a Light Emitting Device

Several applications can be suggested when LENS is integrated as a light source into a relevant module. The following are several conceptual propositions. Since the Advanced Laboratory of Electro-Optics (ALEO) team [18] develops a lot of nanoscale devices—among them LENS—the idea of combining a few of them as modules becomes a desirable and feasible need. In this spirit, developing of a full scale photonic integrated circuit (PIC), i.e., developing an integrated silicon circuitry can bring several combination options. For example, starting from light emitting devices through receivers and converters, one can suggest the following simple solution: including light emitting nano-pixel structures (LENS) [15,16] as radiation sources; including a silicon-on-insulator photo-polarized activated modulator (SOIP^2^AM) [46] and an enhanced optical tunable excited capacitor (EOTEC) [47] as a photonic transistor and capacitor, that realize a Boolean logic Not “OR” (NOR) gate; and finally, including a mechanical-photonic wavelength converter (MPWC) [48] for converting light from infra-red (IR) to visible light and sensing it as an electrical current. Additional combined modules can be suggested, such as Boolean gates like Exclusive “OR” (XOR) or Not “AND” (NAND), generation of a synchronization clock signal, optical memory buffers, multi-spectral sensing devices and more.

## 7. Conclusions

A new dual-mode light emitting nano-pixel structure is proposed and analyzed using complementary studies of its physical properties, compared to previous ray tracing analysis. These new studies, which include a systematic comparison between cylindrical and conical shapes, as well as checks to identify the optimal wavelength, were performed using the Comsol Software package. It is clear that the proposed conical shape enables better results when compared to the regular cylindrical standard shape. The efficiency can be improved even more by setting internal reflective walls inside the conical shape, so that light ray tracing can be optimized.

## 8. Patents

This research is the basis for several future patents.

## Figures and Tables

**Table 2:** Summary of the physical parameters used in geometry study, using Comsol.

Parameters	Parameters Definition	Cylindrical LED	Conical LENS
**Device dimensions and parameters:**			
RU_em_	InGaN Emitting layer Upper Radius	332 nm	302.59 nm
RL_em_	InGaN Emitting layer Lower Radius	332 nm	299.09 nm
AU_em_	InGaN Emitting layer Upper Area	0.346 μm^2^	0.287 μm^2^
AL_em_	InGaN Emitting layer Lower Area	0.346 μm^2^	0.281 μm^2^
D_base_	n-GaN Base diameter	664 nm	45 nm
D_top_	p-GaN Top surface diameter	664 nm	664 nm
OH	Overall Height	442 nm	442 nm
t_em_	InGaN Emitting layer distance from top LED surface	42 nm	42 nm
t_InGaN_	InGaN Emitting layer thickness	5 nm	5 nm
t_sub_	InGaN Emitting layer distance from bottom LED surface	395 nm	395 nm
**Comsol setup used parameters:**			
V_p_	P-GaN applied Voltage	0 V–3 V	0 V–3 V
I_p_	Applied Current	1 × 10^−6^–1 × 10^−3^ A	1 × 10^−6^–1 ×10^−3^ A
p-GaN_up	Doping concentration of p-GaN upper layer	1 × 10^18^ cm^−3^	1 × 10^18^ cm^−3^
n-GaN_lo	Doping concentration of n-GaN lower layer	1 × 10^18^ cm^−3^	1 × 10^18^ cm^−3^
InGaN	Doping concentration of InGaN embedded layer	intrinsic	intrinsic
E_BG InGaN_	InGaN Energy Bandgap for λ = 450 nm (blue)	2.759 V	2.759 V
V_BG GaN 450_	GaN Energy Bandgap for λ = 450 nm (blue)	3.7 V	3.7 V
V_BG GaN 525_	GaN Energy Bandgap for λ = 525 nm (green)	3.4 V	3.4 V
V_BG GaN 650_	GaN Energy Bandgap for λ = 650 nm (red)	3.0 V	3.0 V
